# Automatic bad channel detection in intracranial electroencephalographic recordings using ensemble machine learning

**DOI:** 10.1016/j.clinph.2017.12.013

**Published:** 2018-03

**Authors:** Viateur Tuyisenge, Lena Trebaul, Manik Bhattacharjee, Blandine Chanteloup-Forêt, Carole Saubat-Guigui, Ioana Mîndruţă, Sylvain Rheims, Louis Maillard, Philippe Kahane, Delphine Taussig, Olivier David

**Affiliations:** a Univ. Grenoble Alpes, Grenoble Institut des Neurosciences, GIN, F-38000 Grenoble, France; b Inserm, U1216, F-38000 Grenoble, France; c Neurology Department, University Emergency Hospital, Bucharest, Romania; d Neurology Department, Carol Davila University of Medicine and Pharmacy, Bucharest, Romania; e Department of Functional Neurology and Epileptology, Hospices Civils de Lyon, Lyon, France; f Lyon Neuroscience Research Center, INSERM U1028, CNRS UMR 5292, Lyon, France; g Epilepsy Institute (IDEE), Lyon, France; h Research Center for Automatic Control (CRAN), University of Lorraine, CNRS, UMR 7039, Vandoeuvre, France; i Department of Neurology, Central University Hospital, CHU de Nancy, Nancy, France; j Medical Faculty, University of Lorraine, Nancy, France; k Laboratory of Neurophysiopathology of Epilepsy, Centre Hospitalier Universitaire Grenoble-Alpes, Grenoble, France; l Department of Pediatric Neurosurgery, Fondation Rothschild, F-75940 Paris, France

**Keywords:** Intracranial EEG, Stereo-EEG, Bad channels, Feature extraction, Ensemble bagging, Machine learning, DES, direct electrical stimulation, ECoG, electrocorticography, EEG, electroencephalography, iEEG, intracranial electroencephalography, SEEG, stereo-electroencephalography

## Abstract

**Objective:**

Intracranial electroencephalographic (iEEG) recordings contain “bad channels”, which show non-neuronal signals. Here, we developed a new method that automatically detects iEEG bad channels using machine learning of seven signal features.

**Methods:**

The features quantified signals’ variance, spatial–temporal correlation and nonlinear properties. Because the number of bad channels is usually much lower than the number of good channels, we implemented an ensemble bagging classifier known to be optimal in terms of stability and predictive accuracy for datasets with imbalanced class distributions. This method was applied on stereo-electroencephalographic (SEEG) signals recording during low frequency stimulations performed in 206 patients from 5 clinical centers.

**Results:**

We found that the classification accuracy was extremely good: It increased with the number of subjects used to train the classifier and reached a plateau at 99.77% for 110 subjects. The classification performance was thus not impacted by the multicentric nature of data.

**Conclusions:**

The proposed method to automatically detect bad channels demonstrated convincing results and can be envisaged to be used on larger datasets for automatic quality control of iEEG data.

**Significance:**

This is the first method proposed to classify bad channels in iEEG and should allow to improve the data selection when reviewing iEEG signals.

## Highlights

• We propose a method that detects automatically bad channels from intracranial EEG (iEEG) datasets.
• It computes iEEG features specific to bad channels and uses an ensemble bagging classifier.
• The bad channel classification accuracy was demonstrated to be excellent on a large data sample.

## 1 Introduction

Intracranial electroencephalographic (iEEG) recordings, either from depth electrodes (stereoelectroencephalography, SEEG) (Kahane and Dubeau, 2014) or from subdural grids and strips (electrocorticography, ECoG) (Fernández and Loddenkemper, 2013), are used to localize the epileptogenic zone in some patients with drug-resistant focal epilepsy where other non-invasive measures are limited. These techniques permit to collect prominent data for assessing brain dynamics in pathological and physiological conditions. Particularly, they allow studying human brain connectivity by measuring intracranial responses to direct electrical stimulations (DES) (David et al., 2010, Selimbeyoglu and Parvizi, 2010, Keller et al., 2014). In clinical routine, DES of focal cortical regions is performed using a bipolar derivation of two contiguous contacts and electrophysiological responses are recorded on the remaining contacts, using either ECoG or SEEG. The signals recorded on the two electrodes of stimulation become automatically noisy because the inputs of the amplifier are no longer measuring brain signals.

iEEG recordings thus come with non-neuronal signals from disconnected electrodes during the stimulation procedures, but also with sensors malfunctioning or other parasitic electrical activity recorded by iEEG amplifier and current drift. The channels showing these aberrant signals are classically identified as “bad channels”, after visual inspection by experts. Since bad channels can spoil the quantitative analysis and interpretation of iEEG signals, it is important, specifically for large datasets, to develop methodological approaches that automatically identify them. There are different approaches available in literature that address this challenging task for scalp electroencephalographic (EEG) signals (Shoker et al., 2005, Nolan et al., 2010, Mognon et al., 2011, Lawhern et al., 2013, Alotaiby et al., 2015, Mur et al., 2016). Most often, bad channel detection methods build upon the high spatial correlation of EEG signals and thus predict the value of each channel at each time point from the activity of all other channels at the corresponding time points. A given channel can be considered as bad if it does not correlate with other channels in its neighborhood for a certain threshold. Unfortunately, this assumption is not valid in the case for iEEG signals, where the spatial correlation is much smaller (Nolan et al., 2010).

Despite the great progress made in the literature to automatically detect various types of artifacts from EEG signals, there is still a lack of techniques that specifically handle the problem of bad channels in iEEG data, as to our knowledge no automated method has been published. In practice, the user has to navigate across all stimulation channels to visually identify the bad ones, which has a number of constraints. First of all, this procedure requires a certain level of expertise in iEEG reviewing. Second, this is a tiresome work and it needs a lot of processing time especially for large datasets. Third, it has a poor degree of reproducibility due to intra- and inter-expert variability.

To solve those issues, we propose here a methodological approach that automatically identifies the bad channels from iEEG recordings using ensemble bagging machine learning. This approach includes feature extraction from iEEG signals, training the classifier algorithms and bad channel prediction step. We take the example of SEEG data recorded during DES and used to compute cortico-cortical evoked potentials (David et al., 2013) as we are developing a large multicentric database of such data (f-tract.eu). The method developed here consisted of quantifying several features related to the presence of artifacts from a large number of SEEG files containing DES data. Visual inspection of these data by a SEEG expert allowed to classify the channels as bad or good. From this visual classification, an ensemble bagging classifier was trained and specificity and sensitivity of the machine learning method was quantified using data resampling.

## 2 Methods

### 2.1 Clinical procedure and data acquisition

Epileptic subjects (*n* = 206) used to produce this methodological report have been recorded by five epilepsy surgery units (Grenoble University Hospital – GRE: *n* = 67; Nancy University Hospital – NAN: *n* = 32; Lyon University Hospital – LYO: *n* = 11; Paris Rothschild Foundation – ROT: *n* = 97; Bucharest University Hospital – BUC: *n* = 3). These drug-resistant epileptic patients underwent SEEG recordings and stimulations as part of the presurgical evaluation, in addition to the other standard exams. SEEG acquisitions and DES at 1 Hz were carried out respecting the conformity of conventional procedure applied at each clinical center to properly extract the brain regions to be removed. All the patients gave their written informed consent for their data to be used by the research protocol F-TRACT (INSERM IRB 14-140).

For each patient, 8–17 semi-rigid intracerebral electrodes containing from 5 to 18 contacts of 2 mm length (Dixi Medical, Besançon, France) were implanted, unilaterally or bilaterally, in various cortical areas. SEEG recordings of up to 256 contacts simultaneously were done with a video-EEG monitoring system with a sampling rate of either 256, 512, 1024 or 4096 Hz depending on the cases. One Hz stimulations (pulse width: 1–3 ms) were delivered between contiguous contacts with a rectangular pulse generator of current, using stepwise increasing intensities up to 4 mA or until clinical subjective or objective responses or after-discharges were obtained. Each stimulation run lasted 40 s or less. SEEG data were acquired using a referential montage with the reference electrode chosen in the white matter. A different file was created for each stimulation run, using up to 40 s of baseline before the start of stimulations (and less if the previous stimulation run ended less than 40 s before) and finishing 3 s after the last stimulation pulse. A total number of 10.576 files corresponded to the 206 patients (on average, 51 ± 49 files/patient). A monopolar montage as recorded was used to detect bad channels visually and automatically.

### 2.2 Visual description of bad channels

Because bad channels are supposed to be due to instrumental defaults, all data need to be processed as recorded, *i.e.* using the recording montage configuration, for achieving a correct bad channel labeling. Once it is done, any kind of montage can be used for the processing of the data because no additional bad channels will be “created” by a linear combination of good channels.

**Fig. 1:**
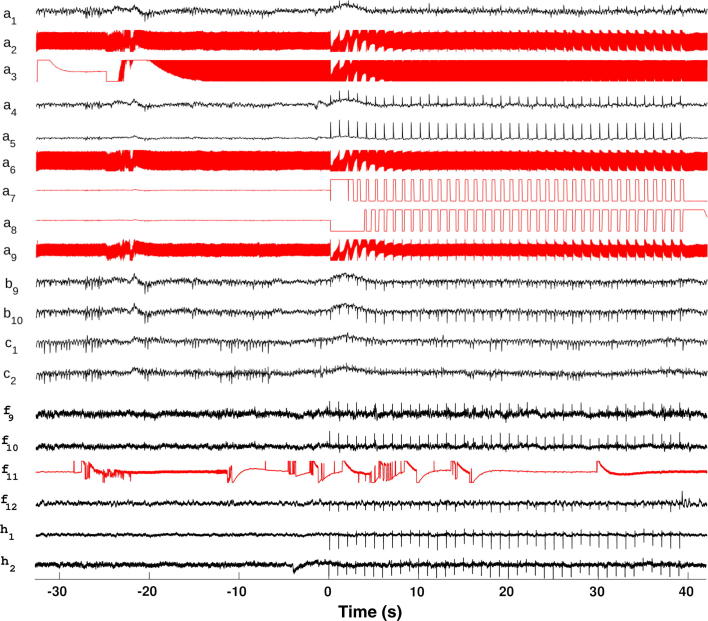
Typical SEEG recording during DES with different observed types of bad channels (in red): (i) channels disconnected from the EEG amplifier (channels a7 and a8); (ii) channels with line noise (channels a2, a3, a6 and a9); (iii) channels with sharp transients due to bad electrical contact (channel f11). (For interpretation of the references to colour in this figure legend, the reader is referred to the web version of this article.)

Bad channels were thus determined by experts using visual inspections of all stimulation files as recorded. Given a stimulation artifact present in a channel recording, different bad channel types could be identified. Fig. 1 shows an example where one can observe: (i) electrode contacts detached from the EEG amplifier (channels a7 and a8); (ii) electrode contacts corrupted with line noise (channels a2, a3, a6 and a9); (iii) channels with intermittent electrical connection (channel f11).

### 2.3 Feature extraction

The computer-assisted approach required a certain number of features to classify bad channels. Here, we selected seven features already used in the literature to remove artifacts from EEG data (Shoker et al., 2005, Nolan et al., 2010, Mognon et al., 2011, Lawhern et al., 2013) that can also be applied to iEEG recordings for detecting bad channels.

i. *Correlation coefficient:* Because of the limited spatial resolution of iEEG, a subset of local electrodes measures spatially correlated brain activity. Therefore, a channel with a low correlation of its activity with the one of its neighbors has an increased likelihood to be bad. The first feature is thus the average of correlation coefficients of every channel with respect to neighboring channels, considered to be on the same electrode shaft, at a maximal distance of 5 contacts. The mean correlation coefficient $\mu_{\mathrm{Cor}}^{i}$ for a given channel $X_{i}$ was computed as follows:
  (1) $$\mu_{\mathrm{Cor}}^{i}=\frac{1}{n}\overset{n}{\underset{j=1}{\sum }}\mathrm{Cor}(X_{i}\text{,}X_{j})$$
  where $\mathrm{Cor}(X_{i}\text{,}X_{j})$ is the Pearson correlation coefficient between channel $X_{i}$ and $X_{j}$, and n the number of channels in the neighborhood of $X_{i}$ (Nolan et al., 2010, Mognon et al., 2011).
ii. *Variance:* A channel with a higher variance with respect to its neighboring channels has also a higher probability to belong to the bad channel class, under the assumption that artefacts add variance to the recorded signals. This feature was also taken into consideration for classification purpose. For this, we computed a normalized channel variance $\sigma_{i}^{\prime 2}$ as follows:
  (2) $$\sigma_{i}^{\prime 2}=\frac{\sigma_{i}^{2}}{{\overset{\sim }{\sigma }}^{2}}$$
  where $\sigma_{i}^{2}$ is the variance for channel $X_{i}$ and ${\overset{\sim }{\sigma }}^{2}$ represents the median of the variances of neighboring channels (Shoker et al., 2005).
iii. *Deviation:* The electrical drift during SEEG recordings can reflect bad channels in terms of electrical impedance. The mean amplitude for a given channel that diverges from the mean amplitude of its neighboring channels can reveal such behavior. It was computed with the following formula:
  (3) $$\Delta_{A}^{i}=\mu_{A}^{i}-\mu_{A}$$
  where $\Delta_{A}^{i}$ and $\mu_{A}^{i}$ are respectively the deviation and mean amplitude of channel i. $\mu_{A}$ is the mean of neighboring channels’ amplitudes (Nolan et al., 2010).
iv. *Amplitude:* The movement of electrodes modulates the impedance between the contact and the electrodes, which in turn alters the offset of electrode voltage. This offset alteration corrupts the channel signal, which is determined by its high amplitude. This signal artifact can be identified by computing the amplitude range normalized by the median of local channels’ amplitudes $\overset{\sim }{A}$ (Shoker et al., 2005):
  (4) $${\text{A}}_{i}^{\prime }=\frac{\text{max}(X_{i}\;)\;-\;\text{min}(X_{i}\;)}{\overset{\sim }{A}}\text{.}$$
v. *Gradient:* The gradient parameter was used to detect high-frequency activity in a channel. For this, we computed the mean gradient of channels using the following formula:
  (5) $$\mu_{G}^{\prime i}=\frac{\mu_{G}^{i}}{{\overset{\sim }{\mu }}_{G}}$$
  where $\mu_{G}^{\prime i}$ is a normalized mean gradient of channel i and ${\overset{\sim }{\mu }}_{G}$ is the median gradient across neighboring channels (Nolan et al., 2010, Mognon et al., 2011).
vi. *Hurst exponent:* The Hurst exponent H is used as an index of long-term memory of time series (Nolan et al., 2010). Typically, EEG recordings have values of H≈0.7, and channels with Hurst exponents that diverge from this standard value are potentially artifacted. In the same line, the Hurst exponent can be applied to detect SEEG bad channels. Given a channel X of length n and mean amplitude $\mu_{A}$, this parameter is computed using the algorithm 1.
vii. *Kurtosis:* An electrical activity may appear in one of the channels and be absent in the remaining ones. Such events can be detected by computing the kurtosis in all channels. Given that the kurtosis indicates the presence of outliers in datasets, the highest value reveals which channel shows a particular event (Mognon et al., 2011).

Note that all feature extraction scripts have been implemented using a commercial software package (MATLAB 9.0, The MathWorks Inc., Natick, MA, R2016a).

**Table:** 

**Algorithm 1.** Calculation of Hurst exponent
1: Calculate the mean amplitude $\mu_{A}$ of channel: $\mu_{A}=\frac{1}{n}\sum_{i=1}^{n}X_{i}$
2: Create a mean centered channel: $Y_{t}=X_{t}-\mu_{A}$ for t=1,2,…,n
3: Compute the cumulative channel deviation $Z_{t}$: $Z_{t}=\sum_{i=1}^{t}Y_{i}$ for t=1,2,…,n
4: Compute the channel amplitude range $R_{n}$: $R_{n}={\text{max}}_{i\in [1\text{,}n]}(Z_{i})-{\text{min}}_{i\in [1\text{,}n]}(Z_{i})$
5: Compute the standard deviation $\sigma_{n}$: $\sigma_{n}=\sqrt{\frac{1}{n}\sum_{i=1}^{n}{\left(X_{i}-\mu_{A}\right)}^{2}}$
6: The Hurst exponent H is given by the following equation: $H=\log{\left(\frac{R_{n}}{\sigma_{n}}\right)}^{1/2}$

### 2.4 Choice of classification model

*A priori*, the datasets have imbalanced class distributions because the number of bad channels is much lower than the number of channels that belong to the class of good channels. Furthermore, the class of bad channels is the class of interest as far as the learning task is concerned. In this case, standard classifier learning algorithms, such as support vector machine, decision trees, logistic regression classifiers, discriminant analysis, and so on, are more sensitive to the finite size of classes in training samples. The degree of weights, for instance in the majority class (here, the class of good channels) is amplified to take advantage of the greater occurrence of instances correctly classified while the instances from the minority class (bad channels) are penalized by low weights as they are usually considered as artefacts. In such a way, bad channels are more often misclassified than good channels (Galar et al., 2012).

Here, we used an ensemble classifier approach to cope with the problem of the class imbalance (Dietterich, 2000). The basic idea of the ensemble methodology is to build different classifiers from the initial data and then to combine their forecasts once new unidentified instances are available. This approach is inspired from the human natural behavior in such way that before any important decision is taken, different consultations had to be gathered. The objective of the ensemble methods is to compile a significant diversity among the individual models they combine and to come up with a new robust classifier that provides more stable and accurate results that outperform each and every single model. The driving principle of combining the predictions of several learning algorithms is to improve generalizability and robustness over a single independent model: for a given test sample, a classifier algorithm that provides a higher classification accuracy will be taken into account with higher weights in that test sample region. Ensemble methods combine many diverse classifiers into a global predictive algorithm in order to reduce variance, bias and thus to improve overall predictions results (Dietterich, 2000, Galar et al., 2012).

### 2.5 Model training and class prediction

We have chosen an ensemble bagging model because of its specific ability to improve classification in terms of stability and predictive accuracy. It also reduces the variance of the classification and helps to avoid overfitting (Galar et al., 2012). The ensemble model used here was implemented in a commercial software package (MATLAB 9.0, The MathWorks Inc., Natick, MA, R2016a). We trained the bagging model using channel features from SEEG datasets, where bad channels were previously labeled by experts. As input, this algorithm took an array of data: channels in raws × 8 columns (the first 7 feature columns and the last column for class labels). After training of the classification algorithm, the prediction is done by applying the trained classifier on new SEEG datasets. The input of the trained model is of the same form as the training dataset (table or matrix) and the trained model returns predictions.

### 2.6 Classification accuracy

The classification performance was assessed using the accuracy rate Acc:

(6) $$\mathrm{Acc}=\frac{\mathrm{TP}+\mathrm{TN}}{\mathrm{TP}+\mathrm{FN}+\mathrm{FP}+\mathrm{TN}}$$

where TP (true positive) is the number of channels labeled as bad and identified as bad channels and TN (true negative), the number of channels annotated and predicted as good channels. FP (false positive) is the number of channels annotated as good identified by the algorithm as bad channels and FN (false negative), the number of channels labeled as bad channels and classified as good.

We evaluated the number of subjects required to obtain a stable classification accuracy. To that end, we trained and tested the classification model with sets of subjects of different sizes (from 10 to 200 subjects for the training set, with a regular step of 10 subjects) randomly chosen among the 206 subjects. For each size of the training datasets, the accuracy rate of classification was computed 19 times.

## 3 Results

### 3.1 Extracted features

**Fig. 2:**
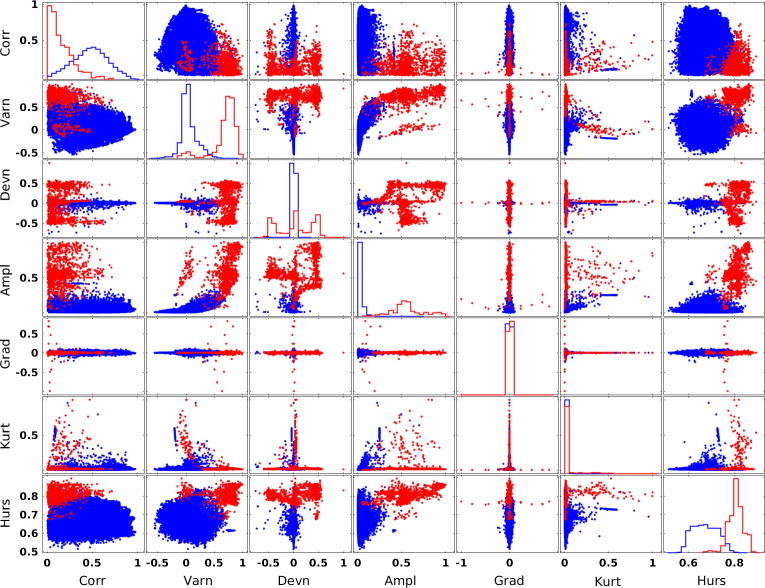
Channels features from 466 SEEG stimulations of 10 subjects. The two channel classes (good channels in blue; bad channels in red) are shown for each pair of features. Features (see Section 2) are: Correlation (Corr); Variance (Varn); Deviation (Devn); Amplitude (Ampl); Gradient (Grad); Kurtosis (Kurt); Hurst exponent (Hurs). Except for Hurs, the amplitude of features was normalized for visualization purpose. (For interpretation of the references to colour in this figure legend, the reader is referred to the web version of this article.)

**Fig. 3:**
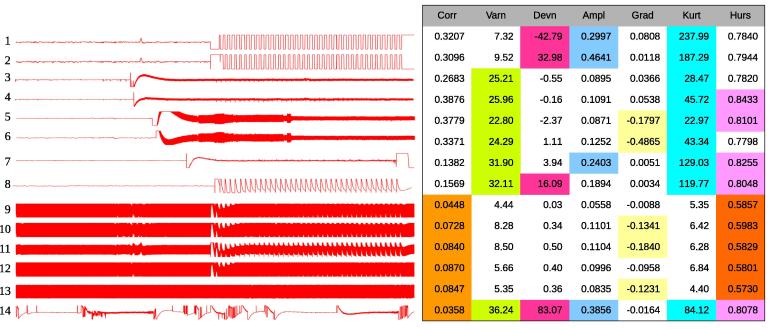
Examples of types of bad channels detected and their respective features: stimulation channels (1:8); channels corrupted by line noise (9:13); channels with intermittent electrical contact (14). The ensemble bagging model combines the most discriminating channel features (some examples of discriminant features for bad channels are highlighted by colors) and makes valid prediction. (For interpretation of the references to colour in this figure legend, the reader is referred to the web version of this article.)

Fig. 2 shows channel features extracted from 466 SEEG stimulations of 10 subjects where the two classes (good channels in blue and bad channels in red) can be visually discriminated. Using these features, ensemble bagging was able to detect different types of SEEG bad channels as depicted in Fig. 3.

### 3.2 Classification accuracy

**Fig. 4:**
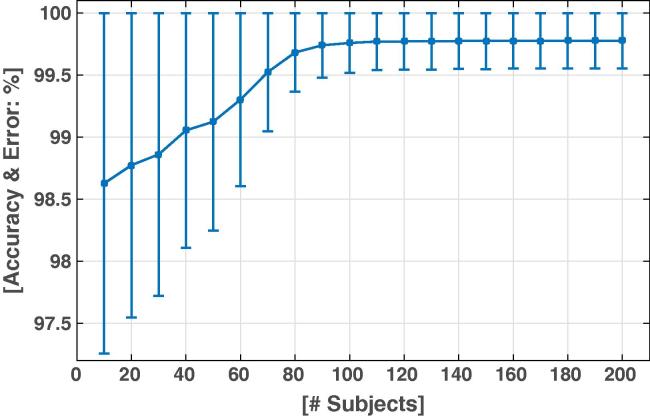
Bad channel detection accuracy and error with ensemble bagging model, as a function of the number of subjects used for the training datasets of the model.

Fig. 4 shows the classification accuracy as a function of the number of subjects used in the training set. With 10 subjects only, the classification accuracy was on average as high as 98.63%. It then increased linearly and reached a plateau at 99.77% for 110 subjects. When more subjects were added to the training set, the accuracy remains unchanged. Therefore, the classification model could be considered as stable and readily usable for any new dataset from there on, with excellent precision. With such high values of classification accuracy, one could anticipate that the classification was robust according to the origin of the data. We explicitly tested this prediction by classifying data coming from centers that were not including in the training dataset. For a training dataset composed of 110 patients, the classification accuracy of the data from each center was very high (BUC: 99.7%; GRE: 99.6%; LYO: 99.6%; NAN: 99.5%; ROT: 99.8%).

### 3.3 Most discriminant features selection

We evaluated the classification ability of each candidate feature to find the most discriminants among others. To achieve this goal, we have used filter methods (Kohavi, 1997) which rely on general characteristics of the data to evaluate and assess the merits of features without taking into account the selected classification model. Under the assumption of no interaction between features, we applied the t-test on each feature and compare *p*-value for each feature as a measure of how accurate it is at classifying bad and good channel groups (Corr: *p* < 0.0001; Varn: <0.0001; Devn: 0.0103; Ampl: <0.0001; Grad: 0.2306; Kurt: <0.0001; Hurs: 0.0078). The computed p-values of all features indicate that all features were discriminant except Grad.

## 4 Discussion

In this paper, an automatic method for the detection of bad channels in iEEG datasets was proposed. It was applied to multicentre large datasets that contained bad channels as found in standard continuous recordings, but also disconnected channels during direct electrical stimulations. The main property of the method is the use of different features and ensemble bagging classifier not only to identify the bad channels but also to cope with datasets with imbalanced class distributions. The results demonstrate a very good accuracy rate (99.77%) and stable classification performances with a training set of about 100 patients and a test set of similar size.

To the best of our knowledge, this report is the first to investigate the possibility to automatically detect bad channels from iEEG data. The methods chosen used up-to-date classification methods from features of interest that were inspired from the methods already developed for scalp EEG (Shoker et al., 2005, Nolan et al., 2010, Mognon et al., 2011, Lawhern et al., 2013). In the implementation of the model for iEEG data as compared to scalp EEG, less emphasis was put on the spatial smoothness of the data as iEEG shows weaker spatial covariance. Different features, *i.e.* Hurst and Devn, were introduced to be sensitive to highly nonlinear signals. However, it should be noted that the presence of the artifact of stimulations in both good and bad channels did not limit the accuracy of the classifier.

Our goal was to develop a method able to deal with data from multiple centers, acquired under different conditions and parameters. We addressed this objective by computing features that are relatively independent of the sampling rate, that is that do not rely in temporal derivatives and frequency contents. By testing the accuracy rate for the patients coming from the different centers, we did not find any significant difference between the centers. This suggests that the classifier can be applied with good confidence to new iEEG datasets coming from different origins.

The proposed method has no profound limitation, except that a training data set has to be prepared by visual inspection of experts. This may be difficult and cumbersome to achieve, but once it is done, the classification model is very easy to use and allows fast classification of channels. One of the advantage of the approach is that the model can be easily incremented with new data in the training set for a larger repertoire of artifacts. This technical note reports early advances of the F-TRACT project (f-tract.eu) for which we collect a large database of cortical stimulations and where an automatic classification of bad channels was required. We thus quantified the accuracy of the methods with the data of the 206 first patients ready to be processed. For the forthcoming patients, the outcome of the model will be systematically controlled visually as part of our quality control procedures. In case of false positive or false negative classification, we will update the classification model. The model is available in open access and will be regularly updated on the F-TRACT webpage (f-tract.eu).

Although the classifier was trained on stimulation data at 1 Hz, the method is also valid to classify bad channels during non-stimulation periods with the same training dataset because many channels did not contain stimulation artifacts and thus could be considered as if recorded during non-stimulation interictal periods. The used training set is however not sufficient to readily take into account stimulation periods performed at other frequencies, such as 50 Hz, the most common frequency in current clinical procedures. In that case, the training dataset will need to be extended to other stimulation protocols to improve the chances of correct classifications for a various set of stimulation configurations.
